# Integrating hydrogeological and second-order geo-electric indices in groundwater vulnerability mapping: A case study of alluvial environments

**DOI:** 10.1007/s13201-021-01437-x

**Published:** 2021-06-25

**Authors:** Nyakno Jimmy George

**Affiliations:** Department of Physics Geophysics Research Group, Akwa Ibom State University, PMB 1162, Mkpat Enin, Uyo, Nigeria

**Keywords:** Geo-electric indices, AVI, GOD, GLSI and longitudinal conductance

## Abstract

AVI (Aquifer vulnerability index), GOD (groundwater occurrence, overlying lithology and depth to the aquifer), GLSI (geo-electric layer susceptibility indexing) and S (longitudinal unit conductance) models were used to assess economically exploitable groundwater resource in the coastal environment of Akwa Ibom State, southern Nigeria. The models were employed in order to delineate groundwater into its category of vulnerability to contamination sources using the first- and second-order geo-electric indices as well as hydrogeological inputs. Vertical electrical sounding technique employing Schlumberger electrode configuration was carried out in 16 locations, close to logged boreholes with known aquifer core samples. Primary or first-order geo-electric indices (resistivity, thickness and depth) measured were used to determine S. The estimated aquifer hydraulic conductivity, K, calculated from grain size diameter and water resistivity values were used to calculate hydraulic resistance (C) used to estimate AVI. With the indices assigned to geo-electric parameters on the basis of their influences, GOD and FSLI were calculated using appropriate equations. The geologic sequence in the study area consists of geo-electric layers ranging from motley topsoil, argillites (clayey to fine sands) and arenites (medium to gravelly sands). Geo-electric parametric indices of aquifer overlying layers across the survey area were utilized to weigh the vulnerability of the underlying water-bearing resource to the contaminations from surface and near-surface, using vulnerability maps created. Geo-electrically derived model maps reflecting AVI, BOD, FLSI and S were compared to assess their conformity to the degree of predictability of groundwater vulnerability. The AVI model map shows range of values of log C ( −3.46—0.07) generally less than unity and hence indicating high vulnerability. GOD model tomographic map displays a range of 0.1–0.3, indicating that the aquifer with depth range of 20.5 to 113.1 m or mean depth of 72. 3 m is lowly susceptible to surface and near-surface impurities. Again, the FLSI map displays a range of FLSI index of 1.25 to 2.75, alluding that the aquifer underlying the protective layer has a low to moderate vulnerability. The S model has values ranging from 0.013 to 0.991S. As the map indicates, a fractional portion of the aquifer at the western (Ikot Abasi) part of the study area has moderate to good protection (moderate vulnerability) while weak to poor aquifer protection (high vulnerability) has poor protection. The S model in this analysis seems to overstate the degree of susceptibility to contamination than the AVI, GOD and GLSI models. From the models, the categorization of severity of aquifer vulnerability to contaminations is relatively location-dependent and can be assessed through the model tomographic maps generated.

## Introduction

In view of the continued rise in population and the challenges of new normal occasioned by the novel corona virus (COVID-19), there is need for integrated assessment of groundwater resources, which serve as good source of potable water over the surface water. To be hygienic, potable water, not just any water, is necessary in this twenty-first century, which demands more use of clean water for survival. According to Sampath (2000), several water wells have been drilled and abandoned mainly due to reasons of defilements caused by infiltration of pollutants through the vulnerably porous lithological layers of the subsurface and the consequent contaminations of water wells instigated by leaching of septic tank, oil spills, refuse dump, naturally and artificially energized corrosions, among other sources. Hydro-geo-resource is intrinsically and specifically susceptible to contaminations. Intrinsic vulnerability connotes an aquifer that is susceptible to pollutions and as well as lithological layering and hydrogeological features. Vulnerability assessment is holistically an essential stride in evaluating groundwater contaminations (Agoubi et al. 2018; Rizka, 2018; George, 2021a). This challenge calls for concerns and the need to scientifically delineate the frequently and economically exploitable hydrogeological units, mostly those that are liable to susceptibility and vulnerability from surface infiltrations in the habitable areas (Vu et al. 2021). As opined by Piver et al. (1997), much pecuniary loss perpetrated by abandonment of well and grave health-related threats would have been obviated if scientific approach that considers well-planned aquifer vulnerability assessment mappings has been opted for as alternative to wildly embraced wildcat drilling. In this era that potable water is highly needed in order to be free from water-borne challenges, assessment of groundwater resources is very necessary in order to identify geologic units that are susceptible to both natural (spontaneous) and artificial (induced) vulnerabilities. Natural vulnerability is a perception that estimates the sensitivity of hydrogeological units to be undesirably affected by an available contaminant burden (Vrba and Zaporozec, 1994). As held by Foster and Hirata (1987), the major parametric indices measured in the natural vulnerability assessment of hydrogeological units include the degree of confinement (open or closed), level of consolidation of the strata overlying the saturated zone, lithological compositions and depth to water table. In general, attenuation of contaminant capacity as well as hydraulic accessibility of the unsaturated zone is the pivot in the assessment of vulnerability (Foster and Hirata 1988). Nevertheless, in Omosuyi (2010) and Aweto (2011) views, hydrogeological units in basement complex environments habitually exist at thin depths and consequently expose the water within it to environmental hazards, which are vulnerable to surface or near-surface pollutants. The safeguarding of the groundwater resources is aided by the covering layers of low coefficient of permeability, which give little or no pathway to percolation of pollutants. This consequently delays and degrades pollutants. Quite a lot of methods have been instituted and used in a systematic way to assess the vulnerability of aquifer recourses to pollutions. A peculiar technique has its merits and demerits. Consequently, there is no known method that can be well-thought-out as the most suitable for a particular situation (Foster et al., 2002; Hassan and Khrisat, 2019). Some of the vulnerability assessment methods include DRASTIC (depth to groundwater, recharge, aquifer type, soil properties, topography, impact of overburden zone and hydraulic conductivity) and confined and unconfined GOD (G = groundwater occurrence, O = lithology of overlying layers and D = depth to the aquifer). These techniques are largely hydrogeological in nature. A few electromagnetic parametric indices like terrain conductivity, longitudinal conductance, which employs geophysical method of measurements, thrive. Some of these methods rely on hydraulic conductivity and thickness of the layers above the aquifer, while others are based on the geo-electric properties of the layers. Known geo-electric method like longitudinal conductance identifies the susceptibility or vulnerability of the geo-electric layer(s). Nevertheless, the results are subject to the principle of equivalence and suppression, which may be insensitive to the existence of comparatively high resistive geological lithology like laterites. Laterites are recognized to be good protective media for the underlying aquifers, and hence, it is essential to adopt other relative techniques such as GOD and geo-electric layer susceptibility indexing (GLSI) in the vulnerability valuation. The GLSI is a recently established method, which intends to overcome the intrinsic weakness of insensitivity to probable existence of lateritic lithology in longitudinal conductance and the over prioritization of the influence of geologic units in the GOD approach. GLSI offers equivalent priority to overburden zone thickness and prominence of lithological units in aquifer safeguarding dynamics by allocating index scores to the layer thicknesses and layer resistivity magnitudes (Oni et al. 2017). The concepts of GOD as opined by Gogu and Dassargues (2000) and GLSI opined by Oni et al. (2017) are index-parametric methods, in which each parameter exhibits a variety of values relating to its property and it is further divided into distinct and hierarchized intervals with explicit values, which reflect their susceptibility level to pollution indices. This paper aims at employing hydrogeological and second-order geo-electric layer susceptibility indices to delineate and categorize geologic layers that are prone to surface or subsurface filterable fluids from contaminated sources.

## Theoretical concept

The conceptual framework in groundwater vulnerability assessment is necessary to characterize the safe and unsafe zones in groundwater resources for proper management and monitoring of groundwater with safety according to requirements of the Water Framework Directive (Jiménez-Madrid et al. 2010). One of the approaches for vulnerability evaluations is the AVI (Van Stempvoort et al. 1992). This method measures vulnerability by hydraulic resistance in years to the vertical flow of water through the shielding/protective layers. Hydraulic resistance is the value that gives the rockability of aquitard to transmit groundwater in a limited amount (Kruseman and de Rider 2000). This value also shows an estimated time for pollutants to pass through the overlaying lithology of aquifer unit pores (Table 1).

**Table 1:** Relationship between *C* and AVI rating (Thomas and Yusrizal, 2018)

Hydraulic resistance C (in years)	Log C	AVI rating
0–10	< 1	Very high
10–100	1–2	High
100–1,000	2–3	Moderate
1,000–10,000	3–4	Low
> 10,000	> 4	Very low

The dynamic process of estimating hydraulic resistance can begin by combination of geo-electric data, measured water resistivity and specific formation constants to estimate the formation-pore hydrodynamic properties, which include effective porosity $(\phi )$ and permeability $(k_{f} )$ that are essential parameters in estimating hydraulic conductivity, *K* (Al-Ismaily et al. 2012; George et al. 2021). From Archie (1942), bulk resistivity $\rho$, water resistivity $\rho_{w}$, geometric factor/electrical tortuosity factor *a*, and cementation factor *m* are related by Eq. 1

1 $$\phi = \left( {\frac{{a\rho_{w} }}{\rho }} \right)^{\frac{1}{m}}$$

Effective porosity for formation grain size of *d* is related to intrinsic permeability in square meter according to Kozeny (1953) in Eq. 2

2 $$k_{f} = \frac{{d^{2} \cdot \phi^{3} }}{{180(1 - \phi )^{2} }}$$

Hydraulic conductivity *K*, used in determining the specific resistance links with permeability using

the Nutting's equation (Hubert, 1940) relates in Eq. 3:

3 $$K = \frac{{\delta_{w} \cdot g \cdot }}{{\mu_{d} }} \cdot k_{f}$$

where $\delta_{w}$ is the water density $(1000\,kgm^{ - 3} )$, $\mu_{d}$ is the coefficient of dynamic friction $(0.0014\,kgm^{ - 1} s^{ - 1} )$ and g is the acceleration due to gravity $(g = 9.81ms^{ - 2} )$. With estimation of hydraulic conductivity, hydraulic resistance $(C)$ for thickness $(h_{t} )$ of each sedimentary unit above the uppermost aquifer and hydraulic conductivity $(k_{i} )$ of each protective layer to the nth layer is gauged using Eq. 4

4 $$C = \sum\limits_{i}^{n} {\left( {\frac{{h_{i} }}{{k_{i} }}} \right)}$$

The relationship between hydraulic resistance in years and AVI rating according to Thomas and Yusrizal (2018) is given in Table 1.

The *k*-values for sandy lithological units are in the order of $10^{ - 5} - 10^{ - 1} ms^{ - 1}$ and are several magnitudes greater than those of clayey layers $10^{ - 8} - 10^{ - 12} ms^{ - 1}$. Thus, *C* has a dimension of time as *K* has the unit length/time (m/s or m/d) and *h* has dimension of length. As held by Van Stempvoort et al. (1992), *C,* can be used to roughly approximate the vertical travel time of water through the unsaturated layers even though some key parameters controlling the travel time like hydraulic gradient and diffusion are not included in the model. Hydraulic resistance (c) values highlight the rockability of aquitard to transmit groundwater in a limited amount (Kruseman and de Rider 2000; Thomas and Yusrizal 2018). This value also indicates an estimated time for contaminants to pass through the overlaying lithology of aquifer unit pores.

With GOD index, aquifer vulnerability can be calculated by multiplication of the influence of the three parameters, namely groundwater occurrence (G) (confined or unconfined aquifer), lithology of overlying aquifer (O) and depth to the aquifer (D) (Oni et al. 2017) as expressed in Eq. 5

5 $${\text{GOD}}\,{\text{Index}} = G \times O \times D$$

Tables 2 and 3 give the attribution of notes for GOD model parameters and the vulnerability rating indices.

**Table 2:** Attribution of notes for GOD index model parameters (Khemiri et al., 2013)

Aquifer type	Note	Lithology (Ω-m)	Note	Depth to aquifer (m)	Note
Non-aquifer	0	< 60	0.4	< 2	1
Artesian	0.1	60–100	0.5	2–5	0.9
Confined	0.2	100–300	0.7	2–10	0.8
Semi-confined	0.3–0.5	300–600	0.8	10–20	0.7
Unconfined	0.6–1.0	> 600	0.6	20–50	0.6
				50–100	0.5
Aquifer type	Note	Lithology (Ω-m)	Note	Depth to aquifer (m)	Note

**Table 3:** GOD parametric index rating (Foster, 1987)

Vulnerability class	Index rating
Negligible	0.0–0.1
Low	0.1–0.3
Moderate	0.3–0.5
High	0.5–0.7
Extreme	0.7–1.0

According to Oni et al. (2017), GLSI is a groundwater assessment technique that applies the indices of geo-electric parameters created from the electrical resistivity contrast between lithological sequences in the subsurface to assess vulnerability or susceptibility of groundwater resources. GLSI allocates index to each of the first-order geo-electric parameters (resistivity and thickness of a layer). It is different from the longitudinal conductance approach, which uses ratios of the first-order geo-electric parameters (thickness and resistivity of layers). This technique is practical and complementary to other approaches used in vulnerability assessment. Tabulations in Tables 4 and 5 give the assigned values of lithology-based resistivity and thickness, respectively.

**Table 4:** Geo-electric layer susceptibility index (GLSI) rating for resistivity parameters

Resistivity range (Ω-m)	Lithology	Susceptibility index rating
< 20	Clay/silt	1
20–50	Sandy clay	2
51–100	Clayey sand	3
101–150	Sand	4
151–400	Lateritic sand	2
> 400	Laterite	1

**Table 5:** Geo-electric layer susceptibility (GLSI) index rating for thickness

Thickness (m)	Index rating
< 2	4
2–5	3
5–20	2
> 20	1

Given that the first layer resistivity index rating is $\rho_{1r}$,first layer thickness index rating is $h_{1r}$, second layer resistivity index rating is $\rho_{2r}$, second layer thickness index rating is $h_{2r}$, nth layer resistivity index rating is $\rho_{{{\text{nr}}}}$, the nth layer thickness index rating is $h_{{{\text{nr}}}}$ and *N* is the number of geo-electric layers overlying the aquifer, according to Oni et al. (2017), *GLSI* can be calculated using the expression in Eq. 6:

6 $${\text{GLSI}} = \frac{{\left( {\left( {\rho _{{1r}} + h_{{1r}} } \right)/2 + \left( {\rho _{{2r}} + h_{{2r}} } \right)/2 + \left( {\rho _{{3r}} + h_{{3r}} } \right)/2 + \ldots + \left( {\rho _{{{\text{nr}}}} + h_{{{\text{nr}}}} } \right)/2} \right)}}{N}$$

In essence, GLSI adopts the multi-criteria decision analysis (MCDA) technique for the rated parametric indices. On this basis, the parametric indices assigned are normalized by dividing everything by the number of inferred geo-electric layers (*N*) above the aquifer. Classification of vulnerability index rating on the basis of *GLSI* according to Oni et al. (2017) is summarized in Table 6.

**Table 6:** GLSI parametric rating

Index	Vulnerability rating
1.00–1.99	Low
2.00–2.99	Moderate
3.00–3.99	High
4.00	Extreme

Based on Henriet (1976), the longitudinal conductance (S) can be used to unearth the degree of protection that aquifer overlying layer offer to its underlying groundwater- rich hydrogeological units. Its capacity to achieve this is directly proportional to the value of the quotient obtained from layer thickness-to-resistivity ratio (Braga et al. 2006; Obiora et al. 2020) given in Eq. 7:

7 $$S = \sum\limits_{i}^{n} {\left( {\frac{{h_{i} }}{{\rho_{i} }}} \right)}$$

In aquifer systems with overlying layers having high longitudinal conductance, generically greater than 1.0 Siemen, give good shielding effect to the aquifers beneath. Consequently, the higher the thickness of overlying layer, the bigger the penetration time of the contaminants and the smaller the resistivity, the more argillaceous and less permeable the aquifer (Oni et al. 2017). The rating of aquifer protection is given by Oladapo et al. (2004) in Table 7.

**Table 7:** Modified longitudinal unit conductance and its protective capacity rating (Oladapo et al., 2004)

Longitudinal conductance	Protective capacity rating
> 10.00	Excellent
5 .00–10.00	Very good
0.70–4.90	Good
0.20–0.69	Moderate
0.10–0.19	Weak
< 0.10	Poor

## Location and geology of the study area

**Fig. 1:**
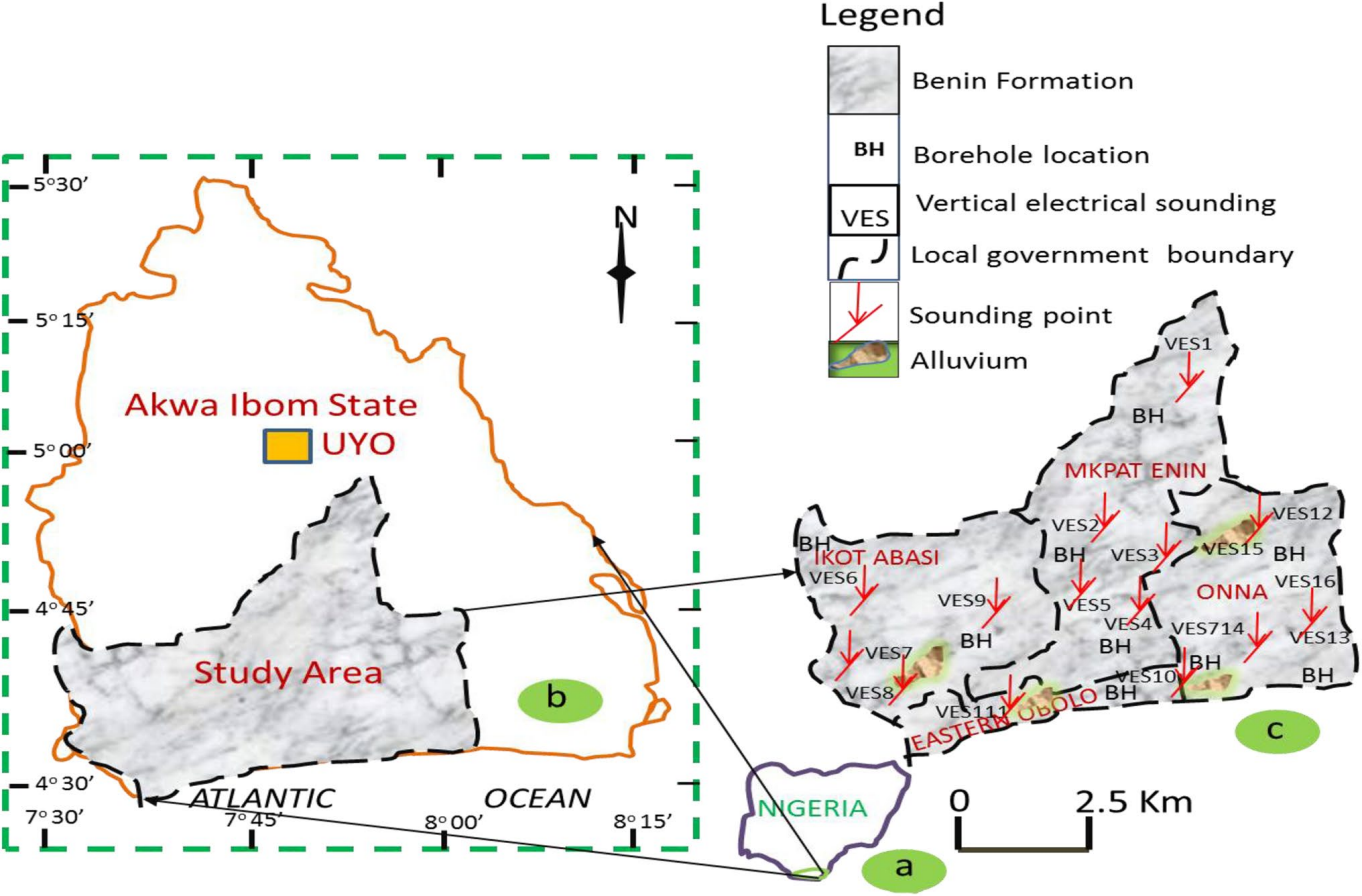
Schematic map of **a** Nigeria showing, the location of **b** Akwa Ibom, which indicate the study area and **c** the study area showing the local geology, VES points, borehole cored sample points, borehole locations and the local government boundaries

The study area is located off the hinterland of the Atlantic Coast of Akwa Ibom State, Southeastern Nigeria (Fig. 1). The study area has an areal extent of about 435 km^2^ in Akwa Ibom State, southeastern Nigeria. Sounding points were stationed within latitudes 4^o^45^'^ to 4^o^35^'^N and longitudes 7^o^30^'^ to 8^o^10^'^ E near borehole points for comfort of interpretation of vertical electrical sounding. The location has average elevation of 102 m above sea level and semi-temperate climate, which defines distinct dry and wet seasons. The wet season lasts from April to September while the dry season occupies the months of October to March. The dry season has a temperature fluctuating from 26 to 32℃. The annual rainfall also fluctuates between 200 and 250 cm. (Uwa et. al. 2019; Thomas et al. 2020). The region was chosen for the hydrogeophysical–hydrogeological study due to the fact that the geological and hydrogeological features of the area were accessible. Specifically, there were existing wells along with well logs providing water samples for measurement of water resistivity and lithological descriptions, which were useful and far- reaching for arriving at geologically consistent results. The geology is underlain by the oldest Eocene to recent Akata Formation (Short and Stauble 1967; Obinawu et al. 2011; Ibuot et al. 2013; Ibanga and George 2016; George, 2021b). This formation occurs as pro-delta facies. Shales of this formation formed at the onset of the Niger Delta Basin development progradation. Overlying the Akata is the younger Agbada Formation that have major reservoir and cap rock for crude oil depositories. This formation is a paralic deltaic front facies with maximum thickness of about 4 km with alternate sands, silts and shales, arranged with successions of about 10—90 feet. The youngest Coastal Formation, which is also known as Benin Formation, overlies the Agbada Formation and its geologic units are mainly brownish and believed to be developed from moderately coarse textured alluvium. Customarily, this geologic formation has characteristic grayish brown and slightly finer to coarser textures that are intermittently intercalating (Short and Stauble 1967; Akpan et al. 2013; Ibanga and George et al., 2016).

## Materials and Methods

**Fig. 2:**
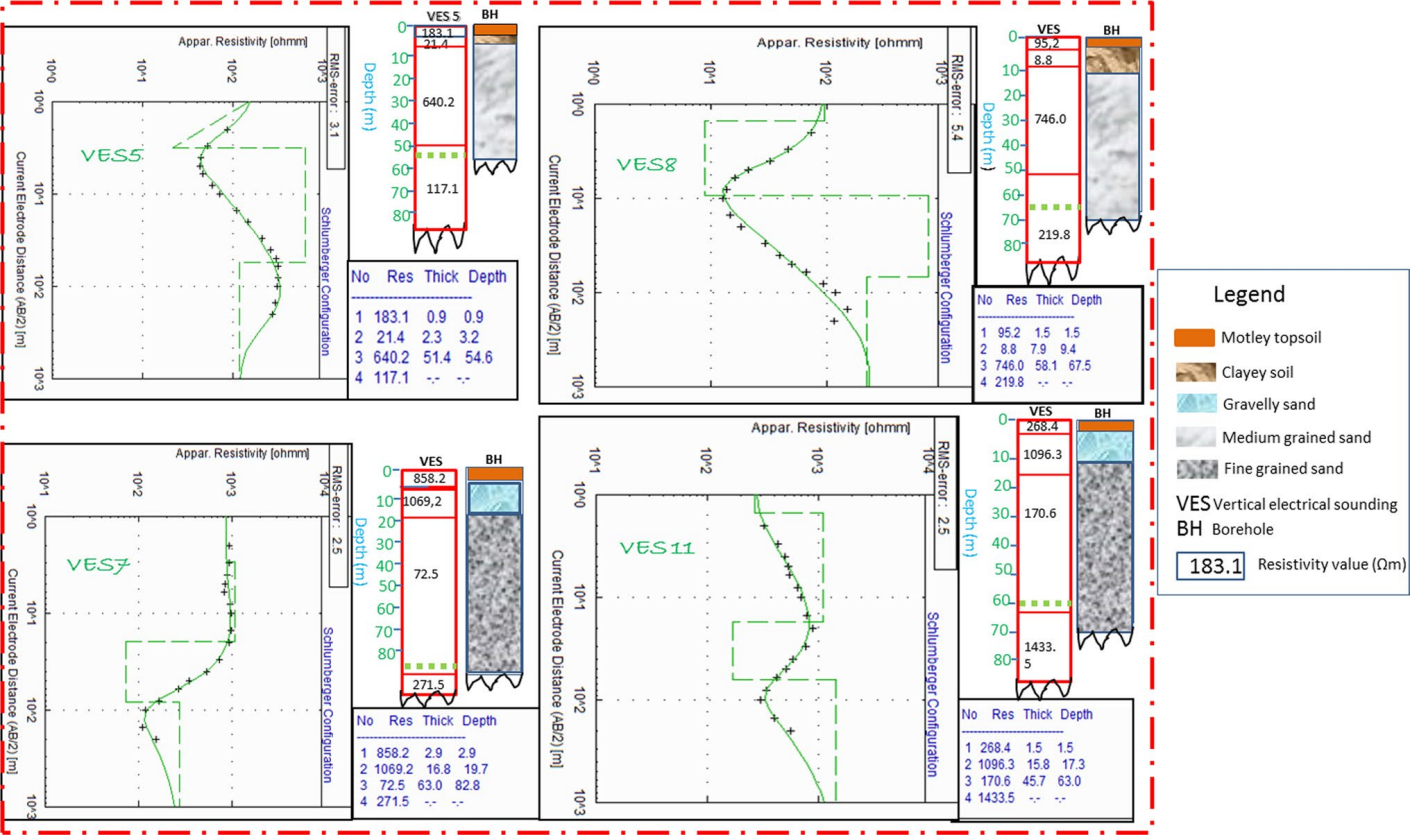
Correlation of VES curves and the nearby lithology formation in the study area (Thomas et al. 2020)

Ground-based geophysics involving 1-D vertical electrical sounding (VES) method was performed and interpreted by constraining them by logged borehole information. This method of geophysical prospecting technique is widely employed in solving hydrogeological and other environmental issues relating to aquifer potential and vulnerability to contaminations (George et al. 2014, 2017; Obiora et al. 2015; Ekanem et al. 2019; Obiora and Ibuot, 2020). From Fig. 1, sixteen VES data were performed near logged boreholes in the survey area in order to have interpreted results that are consistent with geology. As geophysical work needs good field data acquisition, expertise in geophysical interpretations, it is a good practice not to automatically accept results without evidence-based judgment on its geologic plausibility. On this note, to comfortably process the electrical resistivity data, VES interpretations were complemented by logged data as constraints for interpretation of vertical and horizontal units with consistency to geology (Obiora et al. 2015; George et al. 2017; Ibuot et.al. 2019). The Schlumberger electrode configuration technique widely documented in literature (Obiora et al. 2015; George et al. 2017; George and Ekanem, 2019; Ekanem, 2020; Obiora; Ibuot et.al. 2020; Thomas et al. 2020) was applied to assess the distribution of first- and second-order geo-electric indices, using IGIS signal enhancement resistivity meter. Despite the ability of the resistivity meter to average up to 32 cycles of values, measurement cycles were truncated after four stacks, as long as the reading on the liquid crystal display (LCD) had a good match with standard deviation (< 10%). Half of the current electrode separation (AB/2) ranged between 1 and 150 m and that of the potential electrode (MN/2) varied from 0.25 to 30 m. To ascertain strong input signal strength, variation in the potential electrode separation was employed at regular intervals. With the aide of RESIST code, the field data were reduced to their equivalent geological models using computer-modeling techniques (Zohdy, et al. 1974). Following a couple of iterations, a reasonably acceptable variation observed between the field and theoretical data was realized through absolute root mean square (RMS) error, which was commonly found to be less than 10% (Fig. 2). The log-based interpretation of the first-order geo-electric parameters were considered adequate in quantifying the degrees of vulnerability in the area using AVI, GOD and GLSI and unit longitudinal conductance (S). In order to obtain hydraulic resistance, the water resistivity was estimated in situ water electrical conductivity meter. The meter terminals were inserted into water. To measure the conductivity, the button was pressed and the liquid crystal display recorded the conductivity of water. For quality assurance, several readings were performed in each borehole location and the mean value was taken and converted to water resistivity $\rho_{w}$ based on the conductivity $(\sigma_{w} )$—resistivity $(\rho_{w} )$ inverse relation $\left( {\rho_{w} = \frac{1}{\sigma }} \right)$.

## Results and discussion

By applying the ground-based electrical resistivity technique complemented by logged information from nearby boreholes in the study area (Fig. 1); four layers were delineated in the four local government areas in 15 VES points while one VES point at Onna had three layers penetrated by current at its maximum electrode separations (Table 8). Fifteen out of sixteen VES locations defined the layers penetrated with full details (layer resistivity and thickness and depth) while the fourth layer was only defined by resistivity, the reason why the fourth layer is not considered in this investigation. The VES with three layers had two layers fully defined and hence its third layer is not considered in this survey. However, for vulnerability studies, only materials overlying the aquifer layers are considered. Table 8 gives the details of the geo-electric parameters delineated and the summary of range and mean values of resistivity, thickness, depth as well as the VES curve signatures that give rise to the spread of curve types (A, KQ and QH accounting for 6.25% each; HK and HA covering 12.5% each and KH dominating with 56.5%). The curve spreads identify that the geologic units are generically characterized by high and low resistivity reflecting fine to medium sands with minor intercalations with argillites (Ibanga et al. 2016). The correlations of some of the inferred results with nearby lithological logs of borehole are given in Fig. 2.

**Table 8:** Summary of geophysics survey in the study area

VES	Location	Coordinate degree		No of layer	Layer resistivity (Ohm-m)				Layer thickness (m)			Layer depth (m)			Curve type
		Lat	long		ρ_1_	ρ_2_	ρ_3_	ρ_4_	Δx_1_	Δx _2_	Δx _3_	D_1_	D_2_	D_3_	
1	Mkpat Enin	4.7752	7.7854	4	666.5	3606.6	1054.6	375.7	5.3	24.9	32.4	5.3	30.2	62.6	KQ
2	Mkpat Enin	4.7345	7.7733	4	401.6	1108.4	481.9	1195.9	1.1	27.5	50.5	1.1	28.6	79.1	KH
3	Mkpat Enin	4.7034	7.8758	4	395.7	1567.1	510.3	1808.6	0.6	7.9	49.3	0.6	8.5	57.8	KH
4	Mkpat Enin	4.6067	7.8167	4	660.4	1488.6	549.2	1248.7	1.3	3.6	47.8	1.3	4.9	52.7	KH
5	Mkpat Enin	4.6535	7.7332	4	183.1	21.4	640.2	117.1	0.9	2.3	51.4	0.9	3.2	54.6	HK
6	Ikot Abasi	4.6984	7.5511	4	949.9	2715.5	1464.5	3745.2	0.5	3.9	16.1	0.5	4.4	20.5	KH
7	Ikot Abasi	4.6117	7.6317	4	858.2	1069.2	72.5	271.5	2.9	16.8	63.0	2.9	19.7	82.8	KH
8	Ikot Abasi	4.5767	7.5684	4	95.2	8.8	746.0	219.8	1.5	7.9	58.1	1.5	9.4	67.5	HK
9	Ikot Abasi	4.6184	7.7086	4	718.4	1844.9	1362.2	1440.8	0.5	1.6	80.8	0.5	2.1	82.9	KH
10	Eastern Obolo	4.5184	7.8757	4	1433.0	210.0	860.5	8367.3	19.4	43.1	49.6	19.4	62.5	112.1	HA
11	Eastern Obolo	4.5453	7.6359	4	268.4	1096.3	170.6	1433.5	1.5	15.8	45.7	1.5	17.3	63.0	KH
12	Onna	4.7172	8.0167	4	1721.4	118.2	611.2	4893.0	19.6	56.7	36.8	91.6	76.3	113.1	HA
13	Onna	4.5689	8.0278	4	3455.5	1825.6	393.9	2030.4	0.6	2.0	89.2	0.6	2.6	91.8	QH
14	Onna	4.5997	7.8832	4	1157.0	1318.3	735.6	1585.9	1.1	37.0	47.8	1.1	38.1	85.9	KH
15	Onna	4.6629	8.0098	4	156.4	800.8	407.8	1350.1	1.3	9.1	47.9	1.3	10.4	58.3	KH
16	Onna	4.5871	7.8753	3	563.4	966.1	1115.9	–	1.1	75.1	–	1.1	76.2	–	A
Range					95.2–3455.5	8.8–3606.6	72.5– 1464.5	117.1–8367.3	0.5 –19.6	1.6–56.7	16.1–89.2	0.5–91.6	2.1–76.3	20.5– 113.1	
Mean					855.3	1235.3	698.6	2005.6	3.7	17.3	51.1	8.2	24.7	72.3	

**Fig. 3:**
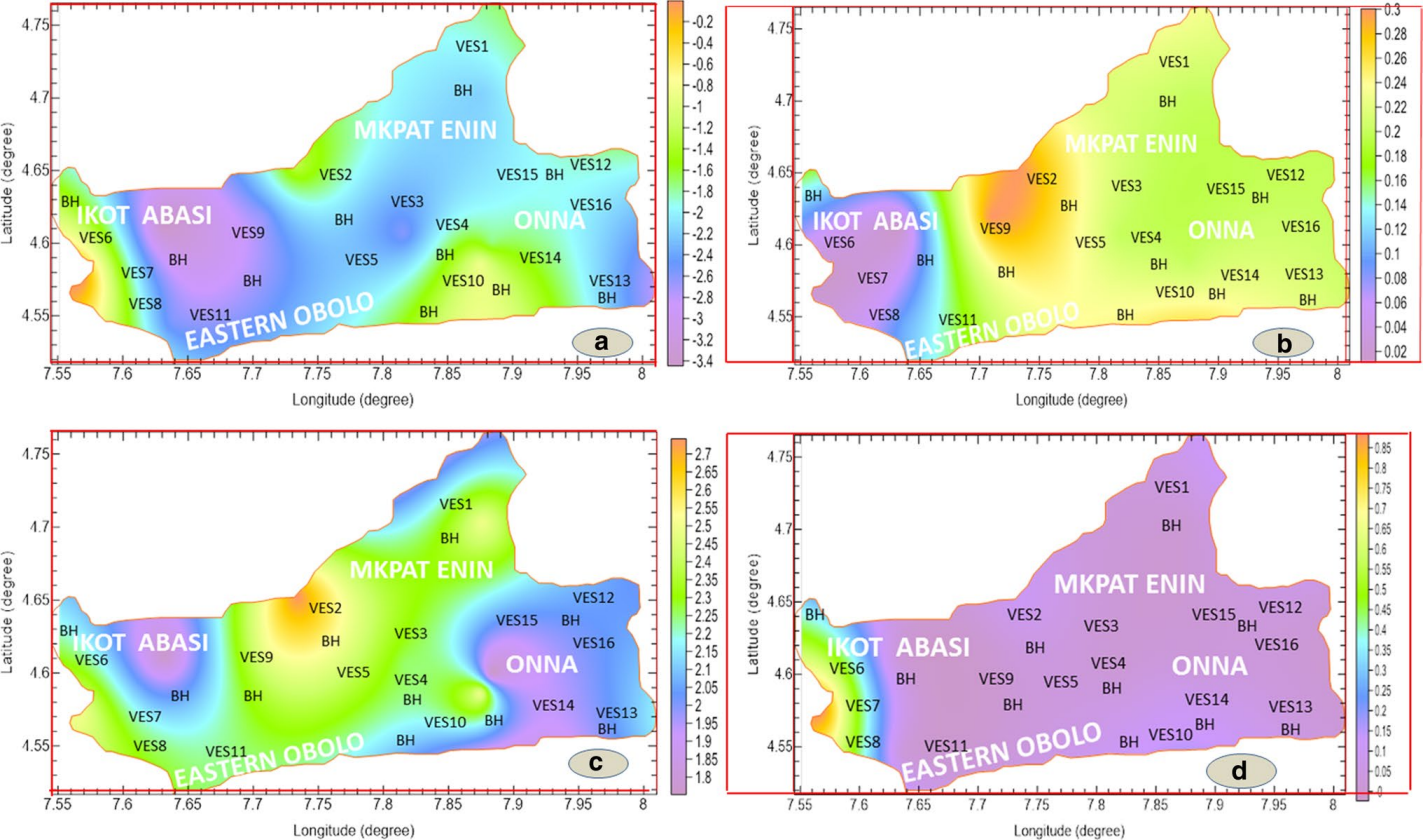
Geo-electric layer susceptible to vulnerability index **a** AVI, **b** GOD, **c** FLSI and **d** S maps

With estimation of effective porosity from measured bulk and water resistivity of the aquifer unit using intrinsic electrical tortuosity parameter and cementation factor $(a = 0.5245\,and\,m_{f} = 1.5430)$, respectively, according to George et al. (2015), hydraulic conductivity was calculated using the relations in Eqs. 2 and 3 for the aquifer geologic units. The aquifer system has mean grain size of $d = 0.000348$ as measured using micrometer screw gauge while its water density was $(\delta_{w} = 1000\,kgm^{ - 3} )$, coefficient of dynamic friction $(\mu_{w} = 0.0014\,kgm^{ - 1} s^{ - 1} )$ and acceleration due to gravity $(g = 9.81ms^{ - 2} )$ (Fetter 1994; Sri and Muhammed 2012; George et al. 2015). With the estimation of aquifer hydraulic conductivity $(K)$ based on its relation with permeability $(k_{f} )$ in Eq. 3, the *K*-values for the two layers above the economically viable aquifers were estimated by congruency, taking into consideration that *K*-value in any geologic unit is congruent to the time rate of conductance of the medium and hence $\left( {\frac{{K_{3} }}{{\rho_{3} }} = \frac{{K_{2} }}{{\rho_{2} }} = \frac{{K_{1} }}{{\rho_{1} }}} \right)$. With 1, 2 and 3 representing layer numbers, the range and mean values for aquifer (layer 3) overlying layers 1 and 2 were estimated to be $2.35 \times 10^{ - 6}$—$1.51 \times 10^{ - 3}$ m/s, $1.33 \times 10^{ - 4}$ m/s and $2.18 \times 10^{ - 7}$—$1.89 \times 10^{ - 3}$ m/s $1.89 \times 10^{ - 3}$ m/s, respectively. Using Eq. 4, the hydraulic resistance, C the AVI was estimated in terms of log(C). Based on the range of log(C) given as (− 3.46–0.07) in Table 9, the study area is highly vulnerable to contamination as log (C) < 1 generally (see Table 1) and image map of Fig. 3a indicates the distribution of log (C) from − 3.46– to .07, indicating various degrees of high susceptibility.

**Table 9:** Summary of inferred ranges of vulnerability indices in the study area

C (years)	Log (C) = AVI	GOD index	GLSI index	S (Siemen)
0.013976	− 1.855	0.3	1.25	0.046
0.013525	− 1.869	0.2	1.75	0.132
0.006543	− 2.184	0.2	2.50	0.103
0.002521	− 2.598	0.2	2.25	0.091
0.054968	− 1.260	0.3	2.75	0.193
0.003369	− 2.472	0.3	2.00	0.013
0.000343	− 3.464	0.1	1.75	0.888
1.172507	0.069	0.1	2.50	0.991
0.002214	− 2.655	0.3	2.50	0.061
0.238019	− 0.623	0.3	1.50	0.288
0.002810	− 2.551	0.1	2.25	0.551
0.248815	− 0.604	0.2	2.00	0.228
0.000723	− 3.141	0.2	2.25	0.094
0.019202	− 1.717	0.2	1.75	0.137
0.014431	− 1.841	0.2	2.00	0.046
0.137483	− 0.862	0.2	2.50	0.132
Range	− 3.46–0.07	0.1–0.3	1.25–2.75	0.013–0.991

From Table 9, the GOD ranges from 0.1 to 0.3 and from Table 3, the study area is generally susceptible to vulnerability, with variability at different locations (Fig. 3b). The GOD image map was formed from the layers above the aquifer and it is considered as index map that combines the effect of distinct layer GOD parameters. The inferred GOD index map generated in Fig. 3b is affected by the inputs of individual GOD constraints. The image map in Fig. 3b identifies of variability of the groundwater vulnerability to contamination in the area under study.

The effect of lithology and layer thickness in aquifer vulnerability assessment is of great importance as sufficiently thick layers above the aquifer can influence by delaying the time of travel of contaminants to the aquifer unit and hence reducing the effect of contaminants in aquifers. These parameters were jointly used to prepare geological layer susceptibility index (GLSI) and its image map in Fig. 3c. GLSI inferred in this work in Table 9 using Eq. 6 and Table 6 has a range of 1.25 to 2.75. The range indicates that the area is susceptibility to contamination with variations from low to moderate rating as indicated in Table 6. With the inferred range, the image map in Fig. 3c indicates the variability of the arrived range for a possible groundwater planning and management in the study area. GLSI is index map of lithological effect and thickness of layers above the considered aquifer.

Generally, longitudinal conductance increases with aquifer protective capacity. As directed in the theoretical concept, the unit longitudinal conductance with average and range values of 0.123 S and 0.003 – 0.913 S were gauged from Eq. 7. The S values indicate that a very small part of the study area has moderate to good protection capacity (0.200—0.913 S) at Ikot Abasi, based on Table 7 estimate and the tomographic image of Fig. 3 d. Outside this location every other point in the study area or the image map shows clusters of very low longitudinal conductance $( < 0.2S)$, adjudged or categorized by Oladapo et al. (2004) to be of poor protection.

Using geophysical and hydrogeophysical parameters, together with assigned indices enabled the estimation of S, AVI, GOD and GLSI models for aquifer vulnerability valuation. The vulnerability indices of each of the concepts obtained from the first- and second-order geo-electric results ease the possibility of creation of vulnerability index maps. The maps generally show susceptibility of aquifer to contaminations with cluster of variations that are not significantly out of the range of poor protective capacity or vulnerability, based on the hydrogeological and geophysical points of view. By relating the AVI, BOD, FLSI and S results on Table 9 and tomographic index maps in Fig. 3, the coastal environment studied can be categorized, in average into zones marked with moderate, low and poor protections. AVI with log(C) < 1 generally suggests that the aquifer is highly vulnerable to contamination from the permeable, inhomogeneous, overlying geologic units. The GOD index, which is less than or equal to 0.3, establishes that the aquifer is negligible to low vulnerability. This claim by GOD model is practically due to the synergistic influence of the bulk lithology and thickness of the overlying layer of the aquifer. The FLSI model suggests that the aquifer assessed is characterized by low to moderate vulnerability based on the protective capacity of the overlying geologic units.

## Conclusion

Ground-based electrical resistivity survey employing VES technique was successfully employed to measure aquifer first-order geo-electric indices, which were further used to estimate the second-order geo-electric indices for vulnerability assessment of coastal environments. The model relies on the synergistic effect of geologic sequence and thickness as the criteria for the gravity of protection offered to any aquifer concerned. Finally, the S model shows that a pocket of the western part of the aquifer under study is replete with moderate to good protection of the aquifer by the overlying geologic medium. However, outside this western part (Ikot Abasi), the rest of the locations have weak to poor protection, indicating high vulnerability to contamination from the overlying units above the aquifer assessed. In principle, the degree of susceptibility/vulnerability is exaggerated by S and AVI than GOD and GLSI models because S and AVI accord greater preference to the geologic lithological thickness than the constituent characteristics of the geologic bed. The degree of vulnerability in GOD is lower than in S and AVI model since GOD gives higher preference to intrinsic properties of geologic units on the basis of geologic unit’s degree of compaction, consolidation, grain size distribution and other inherent attributes that affect the hydrogeophysical, geochemical or geo-electrical properties of a geologic bed (Oni et al. 2017). This study portrays the efficacy of GLSI as significant technique in detecting hydrogeological units that are prone to contamination by virtue of the unique synergistic priority of overlying layer thicknesses. Sufficiently thick aquifer overlying layer could elongate the transit time of descent of contaminants into aquifers underneath. This action delays and consequently degrades the contaminants caused by the synergistic effects of geologic and biogenic activities, thereby making such zones to be minimally susceptible to pollution from associated contaminants. By correlating the bulk specific resistance (AVI-dependent factor) of the aquifer overlying layer and the bulk thickness maps to the assessed models, the entire coastal environment is on the average vulnerable to sources of contaminations due to porous overlying materials above aquifers. On this note, contamination must be anticipated in every borehole dug and hence, underground services must be located away from groundwater sources. Groundwater monitoring, assessment and management must be instituted within the area and its environs with an effort to synergize with the relevant developers and government to reduce the careless dumping of anthropogenic wastes, which finally degrade groundwater resources at large. Regularly monitoring of the level of ingress of contamination reflected by the severity of contaminated water in boreholes must be upheld while efforts should be intensified to measure, treat/disinfect the known contaminated water before consumption.
